# A CT-Based Radiomics Approach for the Differential Diagnosis of Sarcomatoid and Clear Cell Renal Cell Carcinoma

**DOI:** 10.1155/2020/7103647

**Published:** 2020-07-24

**Authors:** Xiaoli Meng, Jun Shu, Yuwei Xia, Ruwu Yang

**Affiliations:** ^1^Department of Radiology, Xi'an XD Group Hospital, Shaanxi University of Chinese Medicine, Feng Deng Road No. 97 Xi'an City 710077, China; ^2^Department of Radiology, Xijing Hospital, Fourth Military Medical University, Changle West Road No. 127 Xi'an City 710032, China; ^3^Huiying Medical Technology Co., Ltd., Room C103, B2, Dongsheng Science and Technology Park, Haidian District, Beijing City 100192, China

## Abstract

This study was aimed at building a computed tomography- (CT-) based radiomics approach for the differentiation of sarcomatoid renal cell carcinoma (SRCC) and clear cell renal cell carcinoma (CCRCC). It involved 29 SRCC and 99 CCRCC patient cases, and to each case, 1029 features were collected from each of the corticomedullary phase (CMP) and nephrographic phase (NP) image. Then, features were selected by using the least absolute shrinkage and selection operator regression method and the selected features of the two phases were explored to build three radiomics approaches for SRCC and CCRCC classification. Meanwhile, subjective CT findings were filtered by univariate analysis to construct a radiomics model and further selected by Akaike information criterion for integrating with the selected image features to build the fifth model. Finally, the radiomics models utilized the multivariate logistic regression method for classification and the performance was assessed with receiver operating characteristic curve (ROC) and DeLong test. The radiomics models based on the CMP, the NP, the CMP and NP, the subjective findings, and the combined features achieved the AUC (area under the curve) value of 0.772, 0.938, 0.966, 0.792, and 0.974, respectively. Significant difference was found in AUC values between each of the CMP radiomics model (0.0001 ≤ *p* ≤ 0.0051) and the subjective findings model (0.0006 ≤ *p* ≤ 0.0079) and each of the NP radiomics model, the CMP and NP radiomics model, and the combined model. Sarcomatoid change is a common pathway of dedifferentiation likely occurring in all subtypes of renal cell carcinoma, and the CT-based radiomics approaches in this study show the potential for SRCC from CCRCC differentiation.

## 1. Introduction

Sarcomatoid renal cell carcinoma (SRCC) is a special subtype of renal cell carcinoma (RCC). Rather than an independent one, it is dedifferentiated from other histological subtypes of RCC both in epithelial and mesenchymal tissues [1]. SRCC is uncommon but highly aggressive, accounting for approximately 1/6 cases of advanced kidney cancers. In particular, it results in more dismal prognosis than the common subtype of clear cell renal cell carcinoma (CCRCC) [2]. According to the newly International Society of Urological Pathology (ISUP) grading system, RCC will be classified to grade IV when a sarcomatoid component was identified [3, 4].

Previous studies report that 45%-84% of SRCC have synchronous distant metastases at the time of diagnosis [5–7]. However, most systemic therapies developed for metastatic RCC are less effective in SRCC [8]. CCRCC can benefit from surgical resection even in the setting of metastasis, while for SRCC patients, surgical resection prior to systemic targeted therapies may worsen the outcomes because it might delay the administration of systemic therapy [2, 9]. Although ablative technique is an option for small renal masses, there is no enough support for using this technique in the small SRCC, and moreover, the infiltrative nature of SRCC tumors makes the determination of negative margin more difficult [2].

Preoperative diagnosis of SRCC is a challenging task. Since recognizable sarcomatoid elements just comprise a variable amount of the whole tumor, the use of biopsy is limited to confirm this entity [10, 11]. Despite some studies that reported that preoperative imaging could be used for predictive diagnosis of SRCC, the small sample sizes of such studies resulted in limited unconvincing consequences [12–15]. In this study, we explore to use of radiomics for the extraction and analysis of high-throughput features and both corticomedullary phase (CMP) and nephrographic phase (NP) images during CT imaging are concerned. Incorporated with clinical information, radiomics could further improve computer-aided diagnosis, prognosis, and predictive accuracy [16–18]. Hence, the purpose of this study is to build a CT-based radiomics approach that uses quantitative features and subjective CT findings for the differentiation of SRCC and CCRCC tumors in a relatively larger sample size.

## 2. Materials and Methods

### 2.1. Data Collection

**Figure 1:**
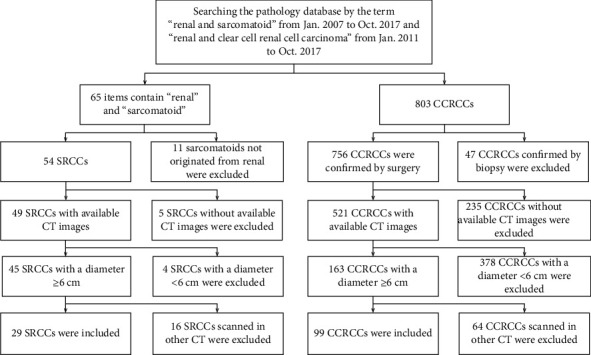
Recruitment pathway for patients in this study.

The study was a retrospective study, and the informed patient consent was waived. Given the predominant number of CCRCC patients and a small number of SRCC patients in our hospital, the SRCC cases were collected from January 2007 to October 2017, and the CCRCC cases were collected from January 2011 to October 2017. To develop a study group with appropriate cases for building the radiomics models, the following inclusion criteria were used: (1) tumors originated from renal; (2) CT with contrast-enhanced CMP and NP images; and (3) tumor diameter ≥ 6 cm. The exclusion criteria were (1) CT images without sufficient quality due to motion artifacts or poor contrast injection; (2) the pathology confirmed as CCRCC only by biopsy; and (3) CT images not acquired in the specified scanner.  Figure 1 shows the recruitment pathway for patient cases in this study.

### 2.2. Clinical Assessment of SRCC and Fuhrman Grades of CCRCC

The determination of SRCC and Fuhrman grade of CCRCC was gathered from the pathology reports, and one pathologist with 8 years of experience specializing in renal pathology reexamined all of the specimens. In this study, one RCC was considered to be SRCC when it resembles any form of sarcoma with or without atypical spindle cells, and a minimum proportion of sarcomatoid tumor was not required to make a diagnosis of sarcomatoid carcinoma. The criterion was in accordance with the ISUP 2012 Consensus Conference [3].

### 2.3. CT Imaging Protocol

The CT images were obtained by the scanner GE Light Speed VCT 64. The scanning parameters were as follows: tube voltage, 120 kVp; the tube current, 250-400 mA using automatic modulation; section thickness, 5 mm; and reconstruction interval, 5 mm. The patients were injected with 1.0 mL/kg of nonionic contrast material (iopromide, Ultravist 370; Bayer, Germany) at rate of 3.5 mL/s via the antecubital vein through a power injector. The CMP and the NP began 25 and 70 seconds after contrast injection, respectively.

### 2.4. Subjective CT Findings

Subjective CT findings for each patient were independently accessed and recorded in a blinded manner by two readers with 6 and 10 years of experience in abdominal imaging, and interreader variability was evaluated by using Kappa statistics. The solution to the divergences for the same case was to ask the readers jointly reviewing it to reach a consensus for further analysis.

Each reader evaluated the tumors for spread pattern, presence or absence of venous thrombus, intratumoral neovascularity, peritumoral neovascularity, calcification, and diameter. (a) Spread pattern was categorized into infiltrative or noninfiltrative. An infiltrative spread pattern was defined as invasion into the collecting system or neighboring organ, or interdigitation into adjacent renal parenchyma with the loss of a clear radiological capsule separating the lesion from adjacent parenchyma. (b) Intratumoral or peritumoral neovascularity means visible vascularity in the tumor parenchyma or perinephric fat adjacent to the mass. (c) Diameter was the largest transverse diameter measured at the maximum axial slice.

The difference in the subjective CT findings between the two patient groups was analyzed by using a chi-squared test or independent sample *t*-test, if appropriate. The findings without significant difference would not be integrated for the model building. All statistical analysis was completed by using SPSS (version 21.0).

### 2.5. Image Segmentation

**Figure 2:**
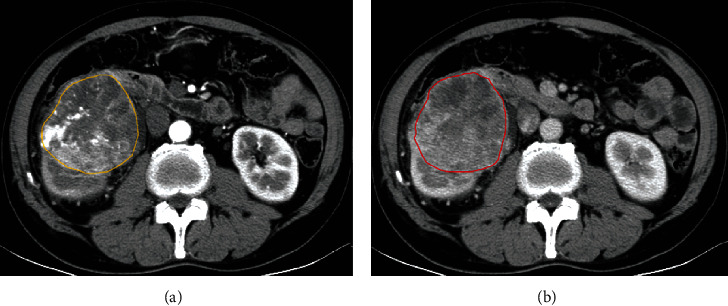
Manual delineation of a SRCC tumor of the same patient at different phases: (a) corticomedullary phase and (b) nephrographic phase.

To obtain the regions of interest (ROIs), the entire tumor of all contiguous slices was outlined except for the first and the last one which aimed to minimize the partial volume effects. Contouring was drawn slightly within the borders of the tumor masses. It included necrotic, cystic change, and hemorrhagic areas, while normal renal tissue and perinephric or sinus fat were excluded. The ROIs were drawn by the two readers both of whom were blinded to the clinical and pathological information.  Figure 2 shows a representative example of manually outlined patient cases.

### 2.6. Radiomics Feature Extraction

To each phase image per patient, 1029 radiomics features were extracted through Radcloud platform (Huiying Medical Technology, http://radcloud.cn/). The radiomics features were divided into first-order features, shape features, and texture features. Shape features were calculated on the original ROI image, while first-order features and texture features were computed on the original ROI image and other derived images obtained by applying several filters, including exponential filter, square filter, square root filter, logarithm filter, and wavelet decomposition [19, 20]. Furthermore, texture features were derived from gray-level cooccurrence matrix (GLCM), gray-level run length matrix (GLRLM), and gray-level size zone matrix (GLSZM). As for the full details of radiomics data, please refer to supplemental S01.

### 2.7. Assessment of Delineation Consistency and Radiomics Feature Stability

To estimate the consistency of delineating CMP and NP images by the two readers, interclass correlation coefficient (ICC) values among 1029 features of each patient and each phase were calculated. If the ICC value of one patient in a phase was greater than 0.75, the manual delineation was considered in good agreement [21, 22] and the delineated image of the first reader would be used in follow-up model construction. Otherwise, the delineation would be repeated by the readers until the ICC met the requirement.

To ensure the stability and reproducibility of the radiomics features, the ICC was also calculated in each radiomics feature between two readers in the CMP and NP images. Features with an ICC greater than 0.75 were regarded as being in good agreement and retained for further radiomics analysis, and others were trimmed off.

### 2.8. Radiomics Feature Selection

Although some of the radiomics features with an ICC lower than 0.75 were removed, there still remained a great quantity of features. In order to decrease the high degree of redundancy and irrelevance, feature selection was conducted using the least absolute shrinkage and selection operator (LASSO) regression method in Anaconda3 platform (https://www.anaconda.com) with scikit-learn (https://scikit-learn.org/) and matplotlib packages (https://matplotlib.org/).

The LASSO regression method has been proved to be efficient and effective in the high-dimensional data analysis [23, 24]. It is aimed at minimizing the cost function and at keeping the features with nonzero coefficients. In this study, features passing the ICC screening were normalized by *Z*-score transform. Then, a 10-fold crossvalidation was carried out to choose the optimal parameters via the minimum of average mean square error. At last, the radiomics features with nonzero coefficients were used for further analysis.

### 2.9. Development, Diagnostic Performance, and Comparison of Classification

In order to evaluate the potential of CT-based radiomics and subjective CT findings for the differentiation of SRCC and CCRCC tumors, 5 models were built with the logistic regression method and fivefold crossvalidation strategy. These models differ from each other, since the selected features are the CMP, the NP, the CMP and NP, the subjective CT findings, and the combined features and subjective CT findings.

*K*-fold crossvalidation is a common model validation technique and widely used in machine learning studies. It randomly partitions the whole set into *K* subsets with equal or close size of data samples. Among the subsets, one is set as the validation set and the others as the training set. The experiment repeats *K* times to ensure that each of the subsets will be used exactly once as the validation set.

Specifically, to build the model with the subjective CT findings, the findings with statistically significant difference were concerned. In this study, infiltrative spread pattern, presence of venous thrombus, neovascularity, and calcification were set as 1, and noninfiltrative spread pattern and absence were set as 0. In the combined model, the CT findings were further selected by Akaike information criterion (AIC) and in the end integrated into a combined model with these selected CMP and NP features.

The quantitative indices used to assess the performance of these classification models were the receiver operating curve (ROC) and the area under the ROC curve (AUC), accuracy, sensitivity, and specificity. The confidence interval of AUC was computed by the exact binomial method. The ROC values of every two models were compared by using the DeLong test [25]. All the model construction, statistical computation, and figures were conducted in the Anaconda3 platform with scikit-learn and matplotlib.

## 3. Results

### 3.1. Clinical Characteristics

This study involved 128 patients (89 males and 39 females; mean age, 57.11 ± 10.52 years; range, 24-80 years). There were 29 (22.66%) SRCC and 99 (77.34%) CCRCC patients. No significant difference was found in gender (*p* = 0.593) or age (*p* = 0.297) between the patient groups, while it showed significant difference in tumor size (*p* < 0.001) and T stage (*p* < 0.001). Patient characteristics are shown in Table 1.

Specifically, among the SRCC patient cases, 23 were dedifferentiated from CCRCC tumors, followed by chromophobe RCC (4 cases), collecting duct carcinoma (1 case), and Xp11.2 translocation RCC (1 case), while among the CCRCC patient cases, the number of Fuhrman I, II, III, and IV was 4, 51, 40, and 4, respectively.

### 3.2. Interreader Agreement of Subjective CT Findings and Radiomics Features

Venous thrombus showed excellent agreement, with a Kappa value of 0.867 (95% CI (confidence interval): 0.598-1.000). Both peritumoral neovascularity and calcification showed good agreement, with Kappa values of 0.629 (95% CI: 0.489-0.761) and 0.787 (95% CI: 0.653-0.901), respectively. Besides, spread pattern and intratumoral neovascularity showed moderate agreement, with Kappa values of 0.571 (95% CI: 0.391-0.733) and 0.404 (95% CI: 0.270-0.537), respectively.

It was found that 1020 CMP radiomics features and 1023 NP radiomics features were with good interreader agreement, and ICC values, respectively, ranged from 0.786 to 0.999 and 0.765 to 0.999. In addition, 9 CMP radiomics features and 6 NP radiomics features were with ICC values less than 0.75, ranging from 0.148 to 0.748 and 0.102 to 0.696, respectively.

### 3.3. The Selection of Subjective CT Findings

It was found out that spread pattern (*p* < 0.001), venous thrombus (*p* = 0.001), peritumoral neovascularity (*p* = 0.017), calcification (*p* = 0.005), and diameter (*p* < 0.001) showed significant differences between the SRCC and CCRCC groups, while there was no significant difference of intratumoral neovascularity (*p* = 0.073) and thus, it was not used in model building. Subjective CT findings between the two patient groups are shown in Table 2.

### 3.4. The Selection of Radiomics Features

**Figure 3:**
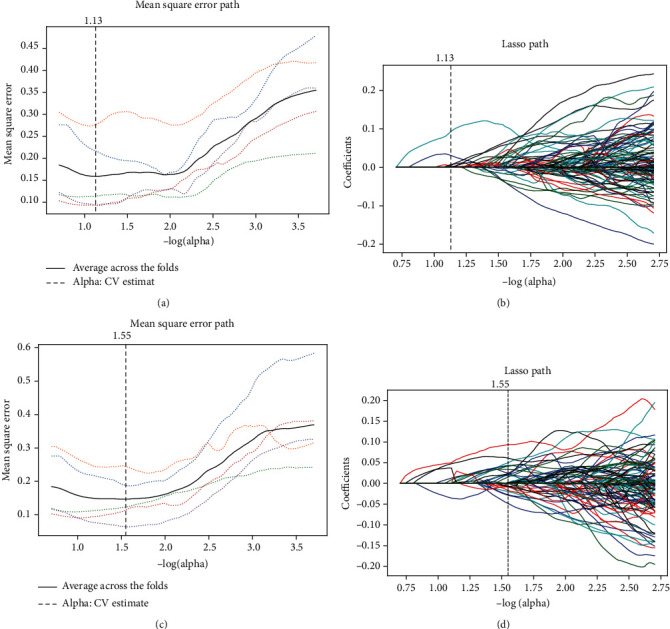
Radiomics feature selection by using the LASSO regression method. The optimal *α* was selected using a tenfold crossvalidation via the minimum of average mean square error. To the CMP features, *α* = 0.074 and −log(*α*) = 1.13 (a) and to the NP features, *α* = 0.028 and −log(*α*) = 1.55 (c). (b) and (d), respectively, showed the coefficient profiles along the full path of possible *α* values in the CMP and the NP feature selection. In addition, dashed vertical lines were drawn at the optimal *α* based on the minimum of average mean square error in (a–d).

Using the regularized regression with the penalty (*α* is denoted as the weight of penalty term), the number of CMP features was reduced to 6 (*α* = 0.074 and−log(*α*) = 1.13) and that of NP features was decreased to 29 (*α* = 0.028 and−log(*α*) = 1.55) with nonzero coefficients. As shown in Figure 3, (a) shows the optimization of the parameter *α* by using LASSO, and (b) indicates the coefficients of selected CMP radiomics features, while (c) and (d) demonstrate the results of the parameter *α* and corresponding coefficients of selected NP radiomics features.

Specifically, the selected CMP features are 3 first-order features, 1 shape feature, and 2 texture features, and the selected NP features include 8 first-order features, 3 shape features, and 18 texture features. The coefficients of selected features are shown in Table 3.

For the combination model, subjective CT findings (spread pattern and calcification) with minimum AIC value were integrated into the selected CMP and NP radiomics features as the input for tumor differentiation. AIC values of subjective CT findings are shown in supplemental S02.

### 3.5. Development, Diagnostic Performance, and Comparison of Classification Models

Five radiomics approaches were explored via logistic regression. The subjective CT findings model considered 4 features (venous thrombus, peritumoral neovascularity, calcification, and diameter). For radiomics approaches, one utilized 6 CMP features, one used 29 NP features, and one concerned these 35 features (6 CMP features and 29 NP features). The last model contained those 35 radiomics features and 2 subjective CT findings.

The diagnostic performance of the five models is shown in Table 4. The subjective CT findings model and the CMP radiomics model showed inferior values of AUC, sensitivities, specificity, and accuracy when compared to the models using NP features, using CMP and NP features, and using the combined features. The CMP radiomics model showed the worst performance with AUC (0.772, 95% CI: 0.689-0.841), accuracy (78.12%), and sensitivity (65.52%), and the combined model achieved the best AUC (0.974, 95% CI: 0.924-0.992), accuracy (93.75%), and sensitivity (96.55%).

**Figure 4:**
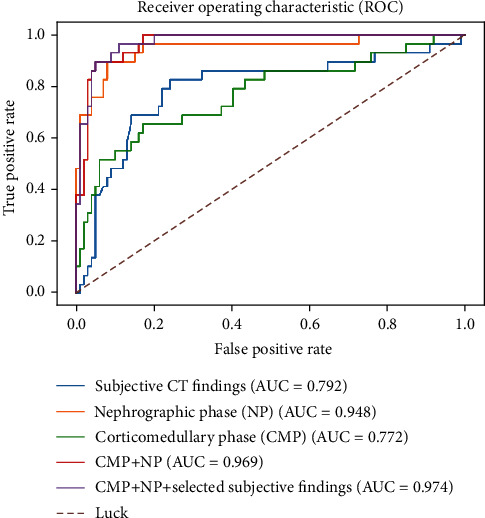
ROC curves of five radiomics approaches for differentiation of SRCC and CCRCC cases. The models are the subjective findings model (blue line), the CMP radiomics model (green line), the NP radiomics model (orange line), the CMP and NP radiomics model (red line), and the combined model (purple line). In addition, the brown dashed line shows the prediction distribution of random inputted features.

Figure 4 shows ROC curves of the five models. The model using combined features achieved the best AUC, followed by the model using the selected CMP and NP radiomics features, and the model using NP features. Relatively, the model using subjective CT findings or CMP radiomics features obtained relatively worse results. According to the DeLong test, there was no significant difference of the AUC values among the NP radiomics model, the CMP and NP radiomics model, and the combined model (0.2245 ≤ *p* ≤ 0.6692), as well as between the CMP radiomics model and the subjective CT findings model (*p* = 0.7479). On the other hand, each of the former three models showed significant improvement compared with each of the latter two models (the CMP model, 0.0001 ≤ *p* ≤ 0.0051; the subjective CT findings model, 0.0006 ≤ *p* ≤ 0.0079).

## 4. Discussion

Sarcomatoid change is believed to be a common pathway of dedifferentiation likely occurring in all subtypes of RCC tumors [4], and preoperative identification of the change is challenging but important in clinic. This study found that the CT-based radiomics approach could help discriminate the SRCC and CCRCC tumors and it also achieved superior performance over the subjective CT findings.

The AUC value using the selected CMP and NP radiomics features was significantly higher than that using the subjective findings, while incorporating the subjective CT findings into the model achieved no incremental predictive value. The AUC value of the NP radiomics model was higher with significant difference than that of the model using the CMP radiomics features. It was slightly lower than that of the CMP and NP radiomics model and that of the combined model with no statistical difference. Such an interesting finding indicated that the NP features are important in radiomics discrimination of SRCC and CCRCC tumors. In addition, the diagnosis power of the NP features better than the CMP features has been reported in machine learning-based CT images [26], which aimed for discriminating fat-poor renal angiomyolipoma from CCRCC. Thus, it might allow the omission of CMP acquisition to reduce the radiation dose in the differentiation of SRCC and CCRCC tumors.

The selected features showed that the “GrayLevelNonUniformity” of the GLSZM texture feature was the most frequently selected feature (Table 3). The feature quantifies the heterogeneity of a tumor. It appeared in the selected SRCC radiomics features with “squareroot_GrayLevelNonUniformity” (coefficient, 0.0926) and in the CCRCC features with “logarithm_GrayLevelNonUniformity” (coefficient, 0.0938) and with “wavelet-HLL_GrayLevelNonUniformity” (coefficient, -0.0063). One reason might be attributed to necrosis which was extremely highly frequent in tumors, for instance, the component of sarcomatoid carcinoma [15], and showed low or nonenhanced in CT images. Some previous studies [27, 28] also highlighted that low enhancement on CT images could be an independent predictor of the presence of high tumor grade of CCRCC, since CCRCC was more heterogeneous [29, 30]. Moreover, that lesion heterogeneity was a feature of malignancy and potential marker of survival, and the patients having heterogeneous tumors with lower uniformity might be with poorer survival [17]. Since SRCC tumors show heterogeneous appearance in multiphase CT imaging, this kind of gray-level nonuniformity feature is difficult to be quantified by a subjective finding until the radiomics emerged.

To the subjective CT findings, they figured out that infiltrative spread pattern, venous thrombus, peritumoral neovascularity, and calcification were more frequently showed in SRCC tumors, yet the Kappa values of these findings were relatively lower. The subjective CT findings to discriminate between SRCC and CCRCC tumors with poor Kappa values were also reported in [14]. However, there is little focus on the calcification of renal mass. One study [30] found that SRCC contained more calcium than RCC (28.6% vs 10.3%) which presumed that calcification is related to necrosis. Indeed, calcification was highly frequent in SRCC, particularly in the components of sarcomatoid carcinoma [15]. In this study, venous thrombus had the highest Kappa value of 0.867, while only 6 out of 29 SRCC and 3 out of 99 CCRCC manifested this feature, indicating the incidence was too low to be used. In short, the subjective findings were valuable but unstable or identifiable in a small cohort, which might account for unsatisfactory diagnostic performance of the subjective CT findings model.

At present, the main research of radiomics approaches for RCC analysis is the renal mass differentiation and nuclear grade prediction [31], and few studies focus on the differentiation of SRCC and CCRCC tumors. To our knowledge, one study explored CT-based radiomics approaches to classify the SRCC and CCRCC tumors [14]. It involved 20 SRCC and 25 CCRCC cases, and both CT subjective findings and texture features were analyzed through noncontrast images. The study indicated that SRCC tumors (7.1 ± 2.7 cm) were significantly larger than CCRCC tumors (5.0 ± 2.9 cm), peritumoral neovascularity and the size of peritumoral vessels differed between the SRCC and CCRCC tumors in the subjective analysis, and SRCC tumors were with greater values of run length nonuniformity and gray-level nonuniformity features. In addition, the classification performance reached an AUC value of 0.81 ± 0.08 based on the combined textural features. Interestingly, as reported in [14], the current study also figured out SRCC tumors with significantly larger size over CCRCC tumors. Except for peritumoral neovascularity, subjective CT findings of spread pattern, venous thrombus, and calcification showed significant difference. In particular, the current study achieved superior performance on tumor differentiation through the analysis of multiphase CT images. It is worth noting that there are two other studies that concerned SRCC and CCRCC tumors by using MRI. One study [15] involved 11 patients with SRCC dedifferentiated from CCRCC tumors, and preoperative renal T1- and T2-weighted MRI were utilized. Compared to a normal renal cortex, it showed that the presence of the areas showing a hypovascular nature and markedly restricted diffusion might be characteristic findings of SRCC. The other study [32] collected 17 patients with SRCC and 17 patients with CCRCC, and dynamic T1-weighted MRI was analyzed. It indicated that the portion of segmented whole tumor with MRI signal suggestive of sarcomatoid involvement was correlated with histological examination, while the percentage of sarcomatoid differentiation was underestimated. Therefore, the current study differs itself from other studies [14, 15, 32] by using multiphase CT.

Multiphase CT was widely used in RCC analysis, and both CMP and NP have been proved to be important in renal lesion differentiation and staging [29]. CMP, the first phase of contrast enhancement, is between 25 and 70 seconds after the injection of contrast material, and the renal cortex enhances more brightly than the renal medulla. NP is the second phase when the contrast material filters through the glomeruli into the loops of Henle and the collecting tubules. At this time, the renal parenchyma becomes homogeneous, and the difference between a normal renal medulla and masses is well observed [33]. In the current study, 6 CMP features and 29 NP features were retrieved, and the NP radiomics approach achieved a significantly higher AUC value over the CMP radiomics approach. The reasons are manifold. First, various amounts of sarcomatoid differentiation are presented in SRCC tumors, which leads to inconsistent CMP imaging features, and in addition, the identified radiomics features cannot well differ the SRCC and CCRCC tumors. Second, NP image features have been reported as the most sensitive features for characterizing CCRCC from other subtypes of tumors, since the features coincided with the maximum tumor-to-kidney contrast [34]. Unfortunately, due to different purposes and specific data sets, there are conflicts of evidence. For instance, [20] indicated that there was no significant difference when CMP and MP radiomics features were independently used for low- and high-grade CCRCC staging, while [35] showed that CMP features resulted in better performance. Therefore, the exact reason why the selected NP features are better than the selected CMP features in the SRCC and CCRCC differentiation requires further investigation.

In the current study, SRCC and CCRCC tumors are with size larger than 6 cm. Two reasons account for this setting. First, one feature differing SRCC from other tumors is their larger tumor size [14]. During the data collection, it was found that almost all SRCC tumors had a diameter larger than 6 cm. Therefore, to reduce the effect of lesion size on the outcome, this study concerned a large tumor size. Second, a large tumor size benefits manual annotation of lesions, and good interreader agreement can be achieved. It should be noted that several studies concerned small RCC tumors. For instance, multiphase CT of tumor attenuation was explored for the differentiation between renal oncocytomas and CCRCC tumors (size ≤ 5 cm) [34, 36] and for distinguishing subtypes of RCC, angiomyolipoma, and oncocytoma tumors (≤4 cm) [21, 37]. Meanwhile, MR image texture features were also utilized for predicting histologic grade of CCRCC with tumor size ≤ 4 cm [38].

There are several limitations of the current study. First, due to the rarity of SRCC, data imbalance occurs. In order to overcome the risk of overfitting, the *K*-folder crossvalidation strategy was performed and the built radiomics approach was verified on an independent data set [39]. To overcome the issue of data imbalance, potential solutions include multicenter collaboration and nationwide and worldwide data sharing. Second, SRCC samples were not stratified according to the underlying diagnosis and the ratio of sarcomatoid component. RCC tumors with even a small component of sarcomatoid change might have an enormously adverse outcome, whereas the primary histologic appearance of SRCC does not have an impact on the prognosis [3, 11]. RCC that contains a sarcomatoid component is categorized to grade IV in the ISUP system, and there was a consensus that a minimum proportion of sarcomatoid tumor was not required to make a diagnosis of sarcomatoid carcinoma [3, 4]. Third, this study concerned SRCC and CCRCC with diameters larger than 6 cm. Pilot studies explored predict histologic grade of CCRCC less than 4 cm using CT and MRI, and statistically significant features were figured out [38, 40], which inspire our future investigation of small RCC samples. Furthermore, MRI features can be embedded into CT-based radiomics approach for improved differentiation [12, 15]. Last but not the least, novel techniques, such as full-automated image segmentation [41], feature dimension reduction [42], multiobjective optimization [43], and deep learning [44], could be further considered for improving classification performance.

## 5. Conclusion

Sarcomatoid change is believed to be a common pathway of dedifferentiation likely occurring in all subtypes of renal cell carcinoma, and preoperative identification of SRCC helps determine the therapeutic strategies. This study shows that the CT-based radiomics approaches could help discriminate the SRCC and CCRCC tumors and further improve patient management, treatment, and quality of life.

## Figures and Tables

**Table 1:** The characteristics of SRCC and CCRCC patient groups.

	SRCC (29)	CCRCC (99)	Whole set (128)	*p* value
Gender				
Male	19 (65.51%)	70 (70.71%)	89 (69.53%)	0.593^a^
Female	10 (34.48%)	29 (29.29%)	39 (30.47%)	
Age (yrs, mean ± std)	55.3 ± 14.0	57.6 ± 9.3		0.297^b^
Size (cm, mean ± std)	10.1 ± 3.0	7.7 ± 1.6		<0.001^b^
T stage				
1b	6 (20.69%)	66 (66.67%)	72 (56.25%)	<0.001^c^
2	11 (37.93%)	19 (19.19%)	30 (23.44%)	
3	11 (37.93%)	13 (13.13%)	24 (18.75%)	
4	1 (3.45%)	1 (1.01%)	2 (1.56%)	

yrs: years; std: standard deviation; *p* < 0.05 is set as significant difference; ^a^*χ*^2^ test; ^b^Student's *t*-test; ^c^Fisher's exact test.

**Table 2:** Subjective CT findings of SRCC and CCRCC patient groups.

Imaging features	SRCC (29)	CCRCC (99)	*p* value
Spread pattern			
Infiltrative	16 (55.17%)	12 (12.12%)	<0.001^a^
Noninfiltrative	13 (44.83%)	87 (87.88%)	
Venous thrombus			
Present	6 (20.69%)	3 (3.03%)	0.001^a^
Absent	23 (79.31%)	96 (96.97%)	
Intratumoral neovascularity			
Present	14 (48.28%)	30 (30.30%)	0.073^a^
Absent	15 (51.72)	69 (60.70%)	
Peritumoral neovascularity			
Present	24 (82.76%)	58 (58.59%)	0.017^a^
Absent	5 (17.24%)	41 (41.41%)	
Calcification			
Present	13 (44.83%)	19 (19.19%)	0.005^a^
Absent	16 (55.17%)	80 (80.81%)	
Diameter (cm, mean ± std)	10.1 ± 3.0	7.7 ± 1.6	<0.001^b^

std: standard deviation; *p* < 0.05 is set as significant difference; ^a^*χ*^2^ test; ^b^Student's *t*-test.

**Table 3:** The selected radiomics features and corresponding coefficients.

Selected CMP features	Coefficients
First-order features	
squareroot_Energy	0.0003
squareroot_Maximum	0.0057
wavelet-LHH_Skewness	0.0069
Shape features	
original_Minoraxis	0.0312
Texture features	
Gray-level run length matrix (GLRLM)	
exponential_RunVariance	0.0037
squareroot_GrayLevelNonUniformity	0.0926
Selected NP features	
First-order features	
wavelet-HLH_Skewness	-0.04831
wavelet-LHH_Median	-0.0272
wavelet-HHH_Median	-0.0076
squareroot_Energy	0.0002
wavelet-LLH_fskewness	0.0019
square_Kurtosis	0.0337
wavelet-LHL_Mean	0.0417
wavelet-LLH_Kurtosis	0.0613
Shape features	
original_SurfaceArea	2.85E-5
original_RunVariance	0.0210
original_SphericalDisproportion	0.0226
Texture features	
Gray-level cooccurrence matrix (GLCM)	
square_Idmn	-0.0196
square_Correlation	-0.0075
wavelet-HHH_ClusterProminence	0.0152
squareroot_DifferenceVariance	0.0422
Gray-level run length matrix (GLRLM)	
wavelet-LLL_ShortRunLowGrayLevelEmphasis	-0.0075
square_ShortRunLowGrayLevelEmphasis	0.0017
wavelet-HHH_RunVariance	0.0225
exponential_RunVariance	0.0242
exponential_RunEntropy	0.0246
exponential_ShortRunLowGrayLevelEmphasis	0.0499
Gray-level size zone matrix (GLSZM)	
square_ZoneVariance	-0.0285
wavelet-HLL_SizeZoneNonUniformityNormalized	-0.0173
wavelet-LLL_LowGrayLevelZoneEmphasis	-0.0086
wavelet-HLL_GrayLevelNonUniformity	-0.0063
wavelet-LLL_ZoneVariance	0.0022
wavelet-HHH_LargeAreaEmphasis	0.0183
logarithm_LargeAreaLowGrayLevelEmphasis	0.0437
logarithm_GrayLevelNonUniformity	0.0938

**Table 4:** The diagnostic performance of the five radiomics approaches.

	AUC (95% CI)	Accuracy (%)	Sensitivity (%)	Specificity (%)
Subjective CT findings	0.792	78.12	82.76	75.76
	(0.712-0.859)			
CMP features	0.772	78.12	65.52	82.83
	(0.689-0.841)			
NP features	0.938	90.62	89.66	91.92
	(0.881-0.973)			
CMP + NP features	0.966	93.75	89.66	94.95
	(0.918-0.990)			
Combined features	0.974	93.75	96.55	88.89
	(0.924-0.992)			

## Data Availability

The clinical CT images used to support the findings of this study are available from the corresponding author upon request, while the radiomics date extracted from CT images is included within the supplementary information file.

## Supplementary Materials

Supplementary Materials S01_Radiomics data: the radiomics data extracted from CT images was included within this supplementary file. S02_AIC radiomics and subjective CT findings: CT findings were further selected by Akaike information criterion (AIC). This supplementary table shows the selected features and corresponding scores. Furthermore, AIC selected features were integrated into a combined model with selected CMP and NP features for tumor differentiation.
