# Supplementary material for: A CT-Based Radiomics Approach for the Differential Diagnosis of Sarcomatoid and Clear Cell Renal Cell Carcinoma
Source: Biomed Res Int. 2020 Jul 24;2020:7103647. doi: 10.1155/2020/7103647 (PMC7397414; doi:10.1155/2020/7103647)
Supplement: Supplementary Materials — S01_Radiomics data: the radiomics data extracted from CT images was included within this supplementary file. S02_AIC radiomics and subjective CT findings: CT findings were further selected by Akaike information criterion (AIC). This supplementary table shows the selected features and corresponding scores. Furthermore, AIC selected features were integrated into a combined model with selected CMP and NP features for tumor differentiation. [file 7103647.f1.zip › S02_AIC radiomics and subjective CT findings.docx]

**Supplementary S03**

**Table S2: AICs about radiomics and** **subjective CT findings**

|  | **Subjective CT findings** | **Akaike information criterion (AIC)** |
| --- | --- | --- |
| \| 0 \| \| --- \| \| 1 \| \| 2 \| \| 3 \| \| 4 \| \| 5 \| \| 6 \| \| 7 \| \| 8 \| \| 9 \| \| 10 \| \| 11 \| \| 12 \| \| 13 \| \| 14 \| \| 15 \| \| 16 \| \| 17 \| \| 18 \| \| 19 \| \| 20 \| \| 21 \| \| 22 \| \| 23 \| \| 24 \| \| 25 \| \| 26 \| \| 27 \| \| 28 \| \| 29 \| \| 30 \| | \| Spread pattern \| \| --- \| \| Venous thrombus \| \| Peritumoral neovascularity \| \| Calcification \| \| diameter \| \| Spread pattern & Venous thrombus \| \| Spread pattern & Peritumoral neovascularity \| \| Spread pattern & Calcification \| \| Spread pattern & diameter \| \| Venous thrombus & Peritumoral neovascularity \| \| Venous thrombus & Calcification \| \| Venous thrombus & diameter \| \| Peritumoral neovascularity & Calcification \| \| Peritumoral neovascularity & diameter \| \| Calcification & diameter \| \| Spread pattern & Venous thrombus & Peritumoral neovascularity \| \| Spread pattern & Venous thrombus & Calcification \| \| Spread pattern & Venous thrombus & diameter \| \| Spread pattern & Peritumoral neovascularity & Calcification \| \| Spread pattern & Peritumoral neovascularity & diameter \| \| Spread pattern & Calcification & diameter \| \| Venous thrombus & Peritumoral neovascularity & Calcification \| \| Venous thrombus & Peritumoral neovascularity & diameter \| \| Venous thrombus & Calcification & diameter \| \| Peritumoral neovascularity & Calcification & diameter \| \| Spread pattern & Venous thrombus & Peritumoral neovascularity & Calcification \| \| Spread pattern & Venous thrombus & Peritumoral neovascularity & diameter \| \| Spread pattern & Venous thrombus & Calcification & diameter \| \| Spread pattern & Peritumoral neovascularity & Calcification & diameter \| \| Venous thrombus & Peritumoral neovascularity & Calcification & diameter \| \| Spread pattern & Venous thrombus & Peritumoral neovascularity & Calcification & diameter \| | \| -207.8151279 \| \| --- \| \| -182.1292789 \| \| -194.3289819 \| \| -194.3289819 \| \| -194.3289819 \| \| -192.3289819 \| \| -192.3289819 \| \| -220.8913564 \| \| -192.3289819 \| \| -180.1292789 \| \| -192.3289819 \| \| -180.1292789 \| \| -192.3289819 \| \| -192.3289819 \| \| -192.3289819 \| \| -190.3289819 \| \| -190.3289819 \| \| -190.3289819 \| \| -218.8913564 \| \| -190.3289819 \| \| -190.3289819 \| \| -178.1292789 \| \| -178.1292789 \| \| -178.1292789 \| \| -190.3289819 \| \| -176.1292789 \| \| -188.3289819 \| \| -188.3289819 \| \| -188.3289819 \| \| -176.1292789 \| \| -174.1292789 \| |
